# A Survey on West Nile and Usutu Viruses in Horses and Birds in Poland

**DOI:** 10.3390/v10020087

**Published:** 2018-02-17

**Authors:** Barbara Bażanów, Petrus Jansen van Vuren, Piotr Szymański, Dominika Stygar, Agnieszka Frącka, Jan Twardoń, Roland Kozdrowski, Janusz T. Pawęska

**Affiliations:** 1 Department of Pathology, Wrocław University of Environmental and Life Sciences, 50-375 Wroclaw, Poland; agnieszkafracka@gmail.com; 2 National Institute for Communicable Diseases of the National Health Laboratory Service, Johannesburg 2131, South Africa; petrusv@nicd.ac.za (P.J.v.V.); januszp@nicd.ac.za (J.T.P.); 3 Faculty of Health Sciences, University of Pretoria, Pretoria 0002, South Africa; 4 The Wild Animal and Bird of Prey Breeding and Protection Center (ZYS Foundation), 54-530 Wroclaw, Poland; p.szymanski.saker@interia.pl; 5 Department of Physiology, School of Medicine with Dentistry Division in Zabrze, Medical University of Silesia, 40-055 Katowice, Poland; dstygar@sum.edu.pl; 6 Department of Reproduction and Clinic of Farm Animals, University of Environmental and Life Sciences, 50-375 Wroclaw, Poland; jan.twardon@upwr.edu.pl (J.T.); roland.kozdrowski@upwr.edu.pl (R.K.)

**Keywords:** West Nile virus, Usutu virus, antibody, Poland, horses, birds

## Abstract

West Nile virus (WNV) and Usutu virus (USUV) are members of the family Flaviviridae which, natural life cycles involve mosquito–bird–mosquito transmission. Both represent emerging viruses in Europe with potential to cause neuroinvasive disease in humans. This study investigates the seroprevalence of serum neutralizing antibodies to WNV and to USUV in birds and in horses in Poland. Antibodies against WNV and USUV were detected in 5 (35.7%) and in 1 (7.14%) of 14 birds and in 62 (15.08%) and in 115 (27.98%) of 411 horses, respectively. Twenty-one WNV serologically positive horses (33.87%) and 67 USUV serologically positive horses (58.26%) did not travel outside Polish borders. Given the high abundance of potentially competent mosquito species in Poland, high populations of horses and different bird species, our findings highlight implementation of active control programs, including monitoring of geographic spread and dynamics of WNV and USUV transmission in both primary and accidental hosts. It is also important to improve public health awareness about the disease these viruses may cause.

## 1. Introduction

West Nile virus (WNV) and Usutu virus (USUV) are mosquito-borne zoonotic agents belonging to the genus *Flavivirus*, family *Flaviviridae* [1,2]. WNV was first isolated in 1937 in the West Nile region of Uganda from a patient suffering a mild febrile illness [3,4]. Over the past decades, the virus became endemic in Europe, Africa, Asia, the Middle East and Australia [5] and it is now well recognized as a human [1,4,5,6], equine [7,8], and avian neuropathogen [1,4,9,10]. In 1999 WNV, was introduced into the United States where it caused severe epidemic of meningoencephalitis in New York City. Subsequently, it spread throughout Canada, Central America and the Caribbean [11]. The occurrence of WNV was confirmed in 20 countries of the European Union, including close neighbors of Poland [1,12]. Previous serological surveys in Poland showed low seroprevalence of WNV antibodies in wild birds, horses and humans [3,12,13,14]. It is rather surprising then that results of recent study in humans indicate high exposure to WNV [14].

USUV was first isolated in 1959 in South Africa from *Culex* mosquitoes [2]. In Europe, it was first isolated in Austria, in 2001, after a significant die-off of Eurasian blackbirds. Subsequently, it has been isolated throughout Europe from countries such as Hungary, Switzerland, Spain, Italy, Germany and Belgium [2]. Serological evidence of USUV presence in birds was also found in England, Czech Republic, Spain, Switzerland, Germany, Italy and Greece. In countries neighboring Poland, such as Germany and Czech Republic, the recurrence of the virus suggests persistence of the transmission cycle in the affected areas, possibly through overwintering mosquitoes [15]. In Poland, antibodies to USUV were found in 2008 in a seagull (*Larus ridibundus*) representing the only evidence of the virus potential activity in the country [13].

The natural life cycles of WNV and USUV involve mosquito–bird–mosquito transmission with occasional spill-over to dead-end hosts such as humans, horses or rodents [2,9,16]. Infected birds develop viremia that allows transmission to competent mosquito vectors. In the world, over 300 bird species may potentially act as vertebrate hosts for WNV [4] and infected migratory birds are thought to spread the viruses to wild birds living in disease free areas [4,11]. Infected birds develop a high-titer viremia that allows transmission to feeding mosquitoes, particularly those belonging to the genus *Culex*, which are considered the principal vectors of WNV. Most infected birds usually survive WNV infection, but certain species have been shown to develop fatal disease [9,10]. Among clinically diagnosed humans and horses, mortality rates can be up to 10% and 25–45%, respectively [4,17]. Human infections due to USUV seem to be less common. The first reported human case was noted in 1981 in Central African Republic in a man with fever and rash [2]. The first human case in Europe was reported in 2009 from Italy and involved a woman with meningoencephalitis [18]. Subsequent studies in humans revealed low seroprevalence to USUV in Germany and Italy [15]. Seroconversion or USUV isolations were reported in birds in many European countries since 2001 [19,20,21,22,23,24,25,26,27,28,29]. Retrospective analysis of material from Italian wild birds found dead in 1996 indicate that the virus was already circulating in Europe at that time [30]. In countries bordering Poland, reports about the presence of USUV neutralizing antibodies in birds were reported from Germany [31,32,33] and the Czech Republic [34]. In 2011, the virus was isolated from a blackbird found dead in Brno, and, in 2012, from two other blackbirds in the same area [35].

We investigated the seroprevalence of serum neutralizing antibodies to WNV and USUV in birds and horses in Poland.

## 2. Material and Methods

### 2.1. Ethics Statement

Clinical specimens from birds were sent to the laboratory for routine diagnosis of viral infections. Horse blood specimens submitted for routine testing for equine viral arteritis were used. Additionally, a part of horse specimens utilized in this investigation was taken during work on other project for which animal ethics clearance for collecting horse blood was obtained from the Wroclaw Animal Experimentation Ethics Committee (No. 43/2011).

### 2.2. Specimens

Tissue samples from birds and blood from horses and birds were collected during the period of October 2012 to April 2013. Tissue samples (brain, liver, lung, heart, spleen, and kidney) were taken from 30 birds representing 10 bird species (Table 1) which died at the Wild Animal and Bird of Prey Breeding and Protection Center in Wroclaw. They were delivered to the Center from different places throughout Poland. Seven birds developed central nervous system symptoms, e.g., abnormal behavior, tremor, head drop or abnormal body posture. Blood for serological testing was collected from 10 healthy goshawks trapped in the field and from four sick birds treated at the Center (three white-tailed eagles, and one common buzzard). The samples were harvested from animals from Lower Silesian, Greater Poland and Łódź voivodeships.

Horses (*n =* 411) were bled on different farms throughout Poland. They were aged 1–28 years old, and included mares (*n =* 251), stallions (*n =* 96) and geldings (*n =* 64) representing polish half-bred (*n =* 74), Arabian (*n =* 42), thoroughbred (*n =* 39), Hucul pony (*n =* 19) Anglo–Arabian (*n =* 15), Malopolski (*n =* 26), Wielkopolski (*n =* 32), Silesian (*n =* 22), polish coldblood (*n =* 37) and Fjord half-bred (*n =* 11) horses. In 94 cases, the breed was unknown. None of the horses were vaccinated against WNV. Although no clinical cases were reported in sampled horses, clinical disease in past is not excluded, because neurological symptoms in horses in Poland are usually diagnosed as equine herpesvirus type 1 (EHV-1) infection or Wobbler syndrome and WNV or USUV infection is not considered [36,37].

### 2.3. Virus Isolation and Identification

Individual tissue samples from birds were processed using a previously described procedure [38] and inoculated separately (50 µL per well, 8 replicates per sample) into different cell lines: rabbit kidney cells (RK-13, ATCC, Manassas, VA, USA, No CCL-37 ^TM^) and green monkey kidney (Vero, ATCC, No CCL-81 ^TM^) seeded in 96-well polystyrene plates the day before inoculation. Plates were incubated at 37 °C/5% CO_2_ and observed daily for the development of cytopathic effect up to 10 days for up to five blind passages. Simultaneously,s virus isolation was attempted in embryonated chicken eggs (ECE). Supernatant from cell cultures and allantoic liquid were harvested and tested by hemagglutination assay and a flavivirus generic RT-PCR, using primers FU1 and CFD2 [39].

### 2.4. Serology

Horse and bird sera were tested for the presence of virus neutralizing antibodies to WNV and USUV using microneutralization procedure as described before [40]. USUV reference isolate SA-AR1776 (South Africa, 1959), Vero passage 4, was used for the USUV neutralization test. The antibody titers were expressed as the reciprocal of the serum dilution inhibiting 100% of viral cytopathic effect. Serum samples with a virus neutralization titer of ≥1:10 were considered positive. IgM serology was performed using the ID Screen West Nile IgM Capture ELISA for equines (IDVet, Grabels, France).

### 2.5. Statistical Analysis

All statistical analysis was conducted in PQStat version 1.6.1 (PQ Stat Software, Poznań, Poland), at a significance level of 5% using the following tests: chi-square and the Spearman rank correlation coefficient (*R*_s_), which statistical significance was then tested by Student’s *t*-test.

## 3. Results

### WNV or USUV Could Not Be Isolated from Any of the Bird Tissue Samples Tested

Virus neutralizing antibodies against WNV were detected in five of the 14 birds tested (35.7%) (Table 1). Of these, three were healthy goshawks: three years old (titer 1:160), one year old (1:80), and 3–4 years old (1:1280). A further two were sick white-tailed eagles: 10 years old (1:20), 4 years old (1:160). Virus neutralizing antibodies against USUV were found in one two years old clinically healthy goshawk (7.14%) (titer 1:10). In one case (one-year-old goshawk), the result could not be concluded since it had neutralizing antibodies against WNV and USUV at the same titer (both 1:10). 

**Figure 1:**
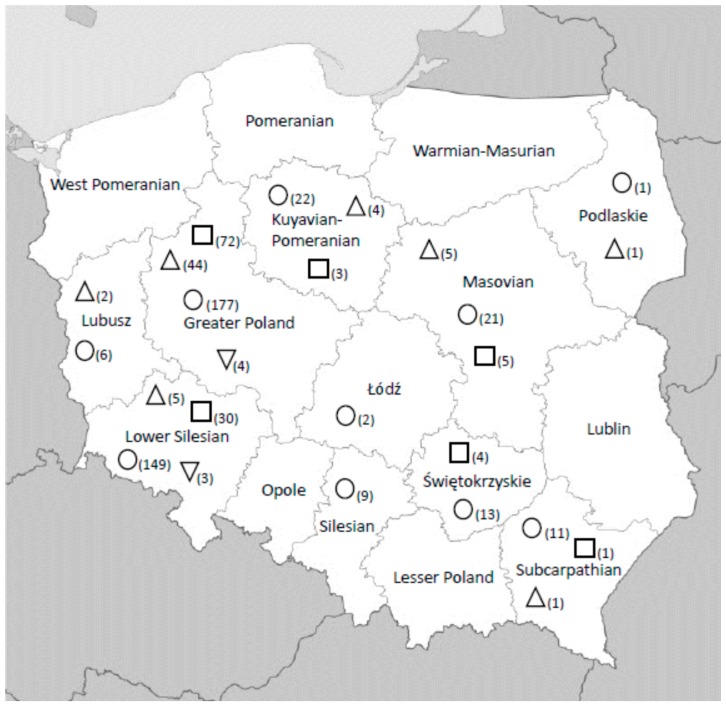
Geographic distribution of WNV and USUV seropositive horses. 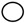 Site and number of sera collected; 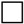 USUV positive serum samples; 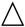 WNV positive serum samples; 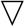 USUV/WNV positive serum samples.

Virus neutralizing antibodies to WNV were detected in 62 horses of 411 tested (15.08%). Seropositive horses were found in different voivodeships of Poland (Figure 1, Table 2). Twenty-one of serologically positive horses (33.87%) did not travel outside Polish borders. The remaining horses traveled to Germany (23 horses), Hungary (9), the Netherlands (4), Lithuania (2), Austria (1), France (1), and Slovakia (1).

Antibodies titers in horses ranged from 1:10 to ≥1:1280: 1:10 (*n =* 2), 1:20 (1), 1:40 (43), 1:80 (1), 1:160 (1) 1:320 (5), 1:640 (4), and ≥1:1280 (5). No WNV IgM antibodies were detected in a subset of 44 horses with confirmed neutralizing antibodies.

Among 411 horse serum samples, 115 (27.98%) were positive for neutralizing antibodies to Usutu virus (Figure 1, Table 2). Sixty-seven of serologically positive horses (58.26%) did not travel outside Polish borders. The travelling horses were in Germany (25 horses), Hungary (6), France (4), Slovakia (4), Austria (3), Italy (3), and the Netherlands (3).

WNV neutralizing antibodies titers in horses ranged from 1:10 to 1:640: 1:10 (*n =* 12), 1:20 (33), 1:40 (29), 1:80 (25), 1:160 (10) 1:320 (4), and 1:640 (3).

In most WNV antibody-positive animals, the USUV titers were negative (virus neutralization titer < 1:10) or significantly lower (0n average two-fold lower) than the corresponding WNV neutralization titers.

In seven horses, the result was positive for both WNV and USUV. One of them (19-year-old mare, titer 1:40) did not travel outside Polish borders. The remaining horses (10-, 13-, 14- and 18-year-old four mares, one one-year-old stallion, and one three-year-old gelding) traveled to Germany (2 horses), Austria (2), Hungary (1) and France (1). The antibody titers reached between 1:10 and 1:40.

Due to the nominal nature of the data, the chi-square test was used to assess the relationship between gender and the WNV or USUV seropositivity, and then between the breed and the WNV or USUV seropositivity. The results showed no significant correlation between gender and the WNV (*p =* 0.212) or USUV (*p =* 0.127) seropositivity and no significant correlation between the breed and the WNV (*p =* 0.861) or USUV (*p =* 0.854) seropositivity.

The association between WNV and USUV seropositivity in individual voivodeships (Table 2) was analyzed using the Spearman rank correlation coefficient (*R*_s_). The results showed no significant correlation between these parameters (*p =* 0.931).

**Figure 2:**
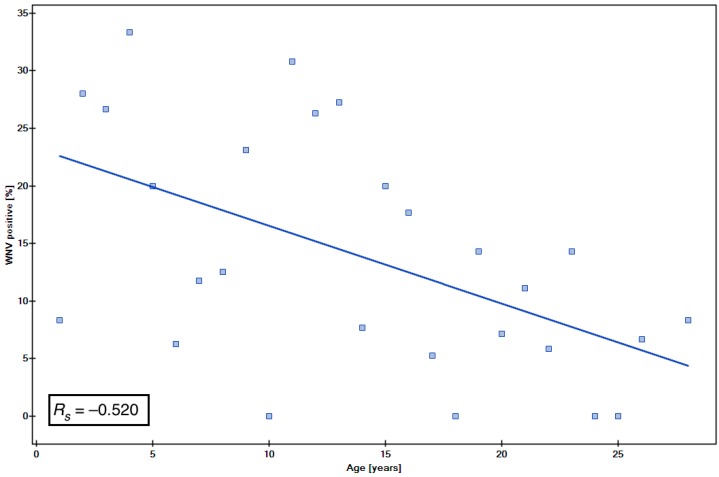
The association between the age and the WNV seropositivity.

**Figure 3:**
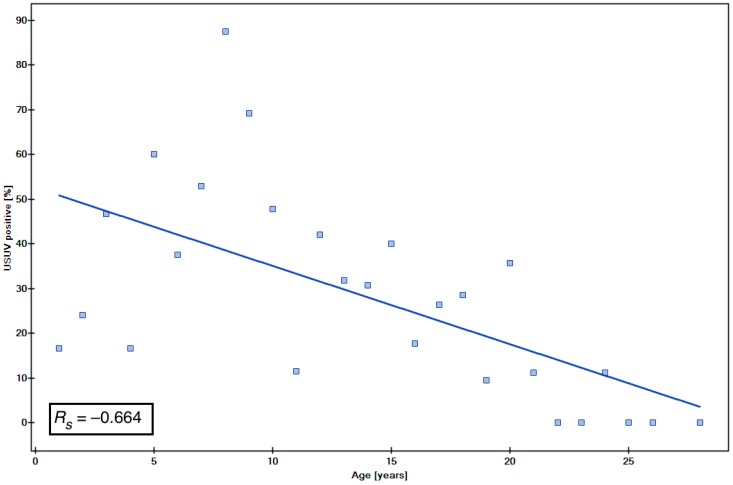
The association between the age and USUV seropositivity.

Due to the not normal distribution of data, to assess the association between the age and the WNV or USUV (Table 3) seropositivity, we also used the Spearman rank correlation coefficient (*R*_s_), the statistical significance of which was then tested by Student’s *t*-test. The correlation coefficient was *R*_s_ = −0.520 for WNV (Figure 2) and *R*_s_ = −0.664 for USUV (Figure 3), which shows a moderate negative correlation between the age and seropositivity. The negative association between age and seropositivity was statistically significant (*p =* 0.005 and *p* < 0.001 respectively).

## 4. Discussion

Earlier studies conducted in Poland showed anti-WNV antibodies in 12.1% of house sparrows and in 2.8% of Eurasian tree sparrows [41], as well as in five out of 97 wild birds (three storks, one crow and one mute swan) [13]. Serosurveys in horses have so far yielded either negative results [13] or a very low level of seropositivity, below 1% [3,14]. In contrast to earlier findings, we demonstrate much higher anti-WNV seroprevalence in both horses (15.08%), and birds (35.7%). Forty-one (66.12%) of the 62 seropositive horses in this study have travelled outside Poland. Therefore, we cannot exclude the possibility that these animals were infected with WNV while travelling outside Polish borders. On the other hand, of 21 VNT seropositive horses without travel history, some animals from different geographic location had very high anti-WNV antibody titers, indicating wide spread local exposure to the virus. Compared to previously published results, the much higher seroprevalence in Polish horses detected in our study might be due to a wider collection of horse sera across the country or it might indicate that in recent years more local horses have been exposed to WNV.

Analysing the percentage of WNV seropositive horses in individual voivodeships, the highest results were observed in Podlaskie (100%) and Lubusz (33.33%), but, **c**onsidering the strength of these groups (1 and 6, respectively), we cannot assume that virus is highly prevalent in these territories. On the other hand, the high ratio in Greater Poland (24.85%), where many horses were tested, may partially come from the fact that many animals from this group travelled abroad. In the case of USUV infection, the high rate was observed in Greater Poland (40.67%) and Lower Silesia (20.13%), where many horses do not move outside Poland. It may indicate that virus circulates in these regions.

A negative association was observed between the age and WNV seropositivity. In the seropositive group of animals that travelled abroad, horses aged 2–13 years old were most represented. Younger horses travel more often to participate in competitions or exhibitions, during which they might be exposed to WNV abroad. Considering the age of horses that did not travel outside Poland, a slightly higher percentage of seropositive individuals was observed in older horses, 16, 21 and 23-year olds (11.11–14.28%).

Considering only animals which did not traveled abroad, the obtained results indicate similar percentage of seropositive horses regardless of gender. In the case of breed, this proportion is also similar (4.55–7.69%) except for Wielkopolski breed, where it is higher (21.88%).

The WNV seroprevalence rate in horses reported in this study is similar or higher than in neighboring countries. For example, in Slovak Republic, the reported seropositivity was 8.3% in 2008–2011, 4.8% in 2012 and 0% in 2013 [42,43]. However, a higher proportion of seropositive horses (13.5%) was detected in 2010–2011 in Ukraine [44].

Antibodies to WNV in birds have been found for over 20 years in Poland [41]. In 2015, Niczyporuk et al. detected VNV antibodies in 13.29% of birds tested [14]. Although we tested relatively small number of birds, our results further confirm circulation of WNV in Polish bird population. Our study included mostly raptor birds and we detected WNV neutralizing antibodies in goshawks and white-tailed eagles. This result is in contrast to the recent study where all the birds with neutralizing antibodies were non-raptors (storks and chaffinches) while raptors were negative [14]. In the case of raptors species, only juveniles migrate whilst looking for a nest site. Adults are sedentary and the highest titers of antibodies against WNV were found in 3–4-year-old birds. In Belarus and Germany, the percentage of WNV-seropositive birds was 6.5–16.7% and 4.6%, respectively [45,46]. The seroprevalence rate in this study was higher and comparable to results obtained in Austria (38.7%) [47].

The high VNV seropositivity found in our study suggests active circulation of the virus in local horse and bird populations. This situation highlights a potential risk for WNV outbreak, including zoonotic transmission to humans. While seroepidemiological data on WNV infection in humans in Poland are limited [48], it is rather worrying that a study conducted in 2015 in Podlaskie voivodeship showed high percentage (33.33%) of patients with meningitis and lymphocytic meningitis to be positive for WNV antigen [14].

We detected anti-USUV neutralizing antibody in one out of 14 goshawks tested. However, his is a second confirmation of USUV seropositivity in birds in Poland. Our study included a very limited number of birds, thus the determination of the extend of USUV infection/transmission in birds in Poland requires further investigations. There is growing evidence for USUV circulation in many bird species throughout European countries [19,20,21,22,23,24,25,26,27,28,29,30,31,32,33,34,35,49].

There are very few reports on USUV infection in horses. In 2013, in Croatia, USUV neutralizing antibodies were detected in two of 69 WNV ELISA-reactive horse serum samples [50] and, in Serbia, in 2011, in one of 349 horses tested [51]. Of 172 horses sampled between 2011 and 2012 on the Island of Mallorca (Spain), 1.2% was seropositive for antibodies against USUV [52]. In Italy, the percentage of USUV seropositive horses was 89.2% in 2008 but only 7.8% in 2009 [53]. 

Our findings suggest a high seroprevalence of anti-USUV neutralizing antibodies in horses in Poland (27.98%) Admittedly, some of these horses (*n =* 48) travelled to countries in which USUV infection has been confirmed, but most of the seropositive animals (*n =* 67) did not travel outside Polish borders.

Since horses appear to be good sentinel animals to monitor USUV circulation [53], our results might indicate that this virus is actively circulating in Poland.

Given the high abundance of different mosquito species from the *Culicidae* family in Poland [54], relatively high population of horses [55], the presence of different bird species, including those migrating between WNV or USUV endemic regions [56] and the results of this study, it is necessary to implement control programs to monitor and prevent WNV and USUV spread and to improve public health awareness of these viruses transmission, and the potentially severe infection these viruses might cause in humans.

## Figures and Tables

**Table 1:** Virological and serological results in birds.

Common Name	Scientific Name	Virus Isolation ^a^	Serology No. Tested/No. Positive		
		No. Tested/No. Positive WNV/No. Positive USUV	WNV	USUV	WNV/USUV
White-tailed eagle	*Haliaeetus albicilla*	10/0/0	3/2	3/0	3/0
Common buzzard	*Buteo buteo*	6/0/0	1/0	1/0	1/0
Goshawk	*Accipiter gentilis*	1/0/0	10/3	10/1	10/1
Peregrine falcon	*Falco peregrines*	1/0/0	n.t.	n.t.	n.t.
Capercaillies	*Tetrao urogallus*	4/0/0	n.t.	n.t.	n.t.
Mute swan	*Cygnus olor*	4/0/0	n.t.	n.t.	n.t.
Saker falcon	*Falco cherrug*	1/0/0	n.t.	n.t.	n.t.
Crossbreed peregrine falcon/gyr falcon	*Falco peregrinus/Falco rusticolus*	1/0/0	n.t.	n.t.	n.t.
European herring gul	*Larus argentatus*	1/0/0	n.t.	n.t.	n.t.
Mallard	*Anas platyrhynchos*	1/0/0	n.t.	n.t.	n.t.

^a^ Virus isolation was attempted from brain, liver, lung, heart, spleen, and kidney tissues. n.t., not tested; WNV, West Nile virus; USUV, Usutu virus.

**Table 2:** WNV and USUV seropositivity in individual voivodeships.

Voivodeship	No. Tested	No. Positive (% Positive)		
		Horses Which Do Not Move Outside Poland	Travelling Horses	Total
Masovian	21	WNV 5 (23.80)	0 (0.00)	5 (23.80)
		USUV 2 (9.52)	3 (14.28)	5 (23.80)
Podlaskie	1	WNV0 (0.00)	1 (100.00)	1 (100.00)
		USUV 0 (0.00)	0 (0.00)	0 (0.00)
Subcarpathian	11	WNV 0 (0.00)	1 (9.09)	1 (9.09)
		USUV 1 (9.09)	0 (0.00)	1 (9.09)
Świętokrzyskie	13	WNV 0 (0.00)	0 (0.00)	0 (0.00)
		USUV 2 (15.38)	2 (15.38)	4 (30.76)
Silesian	9	WNV 0 (0.00)	0 (0.00)	0 (0.00)
		USUV 0 (0.00)	0 (0.00)	0 (0.00)
Łódź	2	WNV 0 (0.00)	0 (0.00)	0 (0.00)
		USUV 0 (0.00)	0 (0.00)	0 (0.00)
Kuyavian-Pomeranian	22	WNV 4 (18.18)	0 (0.00)	4 (18.18)
		USUV 3 (13.63)	0 (0.00)	3 (13.63)
Greater Poland	177	WNV 7 (3.95)	37 (20.90)	44 (24.85)
		USUV 40 (22.59)	32 (18.07)	72 (40.67)
		WNV/USUV1 (0.56)	3 (1.69)	4 (2.25)
Lower Silesian	149	WNV 3 (2.01)	2 (1.34)	5 (3.35)
		USUV 19 (12.75)	11 (7.38)	30 (20.13)
		WNV/USUV (0.00)	3 (2.01)	3 (2.01)
Lubusz	6	WNV 2 (33.33)	0 (0.00)	2 (33.33)
		USUV 0 (0.00)	0 (0.00)	0 (0.00)

**Table 3:** Age of horses tested for WNV and USUV neutralizing antibodies.

Age (yrs)	No. Tested	No. Positive (%)		
		Horses Which Do Not Move Outside Poland	Travelling Horses	Total
1–3	52	WNV 6 (11.53) USUV 7 (13.46)	6 (11.53)	12 (23.07)
			8 (15.38)	15 (28.84)
4–6	43	WNV 1 (2.32) USUV 9 (20.93)	7 (16.27)	8 (18.60)
			8 (18.60)	17 (39.53)
7–9	38	WNV 1 (2.63) USUV 13 (34.21)	5 (13.15)	6 (15.78)
			12 (31.57)	25 (65.78)
10–12	68	WNV 4 (5.88) USUV 16 (23.52)	9 (13.23)	13 (19.11)
			6 (8.82)	22 (32.35)
13–15	50	WNV 4 (8.00) USUV 11 (22.00)	6 (12.00)	10 (20.00)
			6 (12.00)	17 (34.00)
>15	160	WNV 5 (3.12) USUV 11 (6.87)	8 (5.00)	13 (8.12)
			8 (5.00)	19 (11.87)
